# Long-term outcomes of pediatric infections: from traditional infectious diseases to long covid

**DOI:** 10.2217/fmb-2022-0031

**Published:** 2022-03-10

**Authors:** Danilo Buonsenso, Leonardo Di Gennaro, Cristina De Rose, Rosa Morello, Federico D'Ilario, Giuseppe Zampino, Michele Piazza, Attilio L Boner, Cecilia Iraci, Sarah O'Connell, Valentina B Cohen, Susanna Esposito, Daniel Munblit, Joseph Reena, Louise Sigfrid, Piero Valentini

**Affiliations:** ^1^Department of Woman & Child Health & Public Health, Fondazione Policlinico Universitario A Gemelli IRCCS, Rome, Italy; ^2^Center for Global Health Research & Studies, Università Cattolica del Sacro Cuore, Roma, Italia; ^3^Department of Diagnostic Imaging, Hemorrhagic & Thrombotic Diseases Center, Oncological Radiotherapy, & Hematology, Foundation ‘A Gemelli’ IRCCS University Hospital, Rome, Italy; ^4^Pediatric Section, Department of Surgery, Dentistry, pediatrics, & Gynaecology, University of Verona, Verona, Italy; ^5^Patient author, Italy; ^6^Patient author, UK & Northern Ireland; ^7^Patient author, member of the CAC Community Advisory Council of Solve ME/CFS Initiative, Pietro Barilla Children's Hospital, Department of Medicine & Surgery, University of Parma, Via Gramsci 14, Parma, 43126, Italy; ^8^Pietro Barilla Children's Hospital, Department of Medicine & Surgery, University of Parma, Via Gramsci 14, Parma, 43126, Italy; ^9^Department of pediatrics & pediatric Infectious Diseases, Institute of Child's Health, Sechenov First Moscow State Medical University (Sechenov University), Moscow, Russian Federation; ^10^MSc Immunology, Imperial College London, London, UK; ^11^ISARIC Global Support Centre, Centre for Tropical Medicine & Global Health, University of Oxford, Oxford, UK

**Keywords:** children, COVID-19, long covid, microclots, post-COVID-19 condition, SARS-CoV-2, viral persistence

## Abstract

There is limited evidence available on the long-term impact of SARS-CoV-2 infection in children. In this article, the authors analyze the recent evidence on pediatric long covid and lessons learnt from a pediatric post-covid unit in Rome, Italy. To gain a better understanding of the concerns raised by parents and physicians in relation to the potential long-term consequences of this novel infection, it is important to recognize that long-term effect of a post-infectious disease is not a new phenomenon.

## Plain language summary

The authors analyze the recent evidence on pediatric long covid and lessons learnt from a pediatric post-covid unit in Rome, Italy. Also, we analyze the long-term effects of other infectious diseases.

## Tweetable abstract

An analysis of the recent evidence on pediatric long covid and lessons learnt from a pediatric post-covid unit in Rome, Italy.

COVID-19 continues to severely impact global population even after 2 years of official declaration as a public health emergency of international concern. Despite substantial research into SARS-CoV-2 pathogenesis, transmission dynamics and genome evolution, COVID-19-related morbidity and mortality, social restrictions and economical impacts still remain a huge concern [1]. Specifically, there is increasing recognition that a significant number of people have not fully recovered from the initial infection even months later. It is estimated that up to 30% of infected adults are affected by long-term SARS-CoV-2 sequelae, manifesting as a multisystem condition often impacting education and occupation [2]. The long-term consequences of SARS-CoV-2 infection, initially defined as long covid by patient support groups, are characterized by signs and symptoms such as chronic fatigue, cardiovascular problems, coagulation disorders, chronic liver, kidney and lung disease, post-exertional malaise, musculo–skeletal, neurological, cognitive and psychological problems. In October 2021, the World Health Organization (WHO) provided a comprehensive definition of post-covid condition in adults [3]. However, while long covid has been widely described in adults, its burden and characterization are less defined in the pediatric population. In fact, the WHO adds to their guidance that “*a separate definition may be applicable for children*” [3]. There is limited evidence on the long-term impact of SARS-CoV-2 infection in children available. Most studies published to date have focused on adult populations. However, emerging evidence from case studies, patient support groups and clinicians highlight that children worldwide are impacted by long covid. Given the current uncertainties, recent evidence on pediatric long covid and lessons learnt from a pediatric post-covid unit in Rome, Italy, are reviewed. To gain a better understanding of the concerns raised by parents and physicians in relation to the potential long-term consequences of this novel infection, it is important to recognize that post-infectious disease sequelae is not a new phenomenon. Post-viral sequelae has been well described by patients' experiences (Box 1) and in natural history studies (Supplementary Box & Table 1) for well-known infectious diseases in the past [4–57].

Box 1. **Patient experiences**

**An adolescent with long covid**

I'm Cecilia, a 17-year-old girl, born in August 2004, with no relevant pre-existing medical conditions. On 11th April 2021, I woke up during the night having several chills, a bad headache, a high fever and a very severe sore throat. I immediately understood that was not the conventional flu I suffered from in the past, not even similar to the worst one.

I had many debilitating symptoms, such as constant weakness, high fever, shortness of breath, tachycardia, loss of smell and taste and many muscular and skeletal aches, especially in my chest and back. I tested positive for more than 20 days in a row, till the 6th May 2021, when I finally had a negative swab. I felt very sick during my quarantine, and I spent many hours a day sleeping. The unmanageable weakness, tachycardia, frequent headaches, extreme difficulties breathing and aches, persisted even after the assumed healing time. I took an entire month's rest to try to recover completely: I was sure I just needed time to heal perfectly and to be able to go back to a regular and painless teenager's life.

Afterward, in June, the situation was static. I was complaining about not feeling well, and most of all, I felt so scared and lonely as no doctor believed my pain: “*you're a young girl, it's all in your head, covid doesn't affect young people this much*”, they told me. I knew something was going on in my body since I was still feeling very ill; I had many daily pains, I couldn't walk for more than 10 min or go up the stairs without having tachycardia, chest aches and breathlessness. I didn't give up on myself even when I felt all-up; I cried on my mum's chest through entire nights because I wasn't feeling safe and I didn't recognize myself.

In June 2021, I finally met a doctor who diagnosed me with long covid. That diagnosis paradoxically made me pleased, made me feel understood by legitimizing my pain and symptoms. I did many hospital visits and exams to analyze what was going on and inform a medical treatment strategy.

It's been almost a year now since I got COVID-19, and I'm still struggling with pain and difficulties. This current year is my last one in high school. I have to attend many courses to have a chance to get into medical university. It's been very demanding lately: since I got covid, I'm not able to concentrate as well as before. I feel like I'm not capable of thoroughly understanding what I study.

I'm truthfully still trying to figure out how this disease hit my body and changed it. It's very challenging for a teenager to face an infectious disease like this, since we're all still learning about it. I honestly hope this unpredictable and damaging experience will be a terrible nightmare as soon as possible.

**An adult with myalgic encephalomyelitis/chronic fatigue syndrome (ME/CFS)**

Forty years ago, after spending the summer studying English as my second language at a rigorous preparatory school in the USA and graduating with honors, upon returning back home to start my freshman year in high school, I came down with an infection that changed my life forever. A common case of infectious mononucleosis, caused by Epstain–Barr virus (EBV), ripped through my young body and brain, disrupting my ability to study and be my former active self. I have been ill for 40 years now, 30 of which without a proper diagnosis. Ten years ago my health deteriorated to the point of becoming house and bed-bound. The illness that has affected me since that fall, has not only stolen my capacity to use my brain, but also my ability to participate and perform the most basic of everyday life activities. This condition has a long complicated history, made up of neglect, disbelief and mistreatments of millions of sufferers around the world. Its core symptom is called PEM (post-exertional malaise) a vicious physiological payback, a crash, after even minimal physical, mental or emotional activities. The illness has a name: ME (myalgic encephalomyelitis) and as such has been recognized by the WHO, since 1969, as a post-viral neurological disease. Through biomedical research we are starting to recognize it as a neuro-immuno-metabolic, chronic and systemic illness. Many cases start after an infection. People with ME, are for the vast majority still left undiagnosed or more frequently misdiagnosed. Our illness is finally starting to be talked about due to the many clinical similarities with long covid. Our greatest hope is to be finally listened to, believed, diagnosed, counted, properly researched and treated, along with the many who may be at risk of developing ME/CFS after COVID-19. May Dr Oliver Sacks' words be a guiding light and an admonition: “*Our patients are our teachers.*”

**A parent of a child with ME/CFS**

When my daughter first started being unwell, it didn't occur to me that her symptoms could be ME. She was only 7 years old, and her symptoms were initially very mild. She complained that her throat was sore every now and then and she was tired a lot. My mother (her grandmother) and I both have ME, but we both had only become unwell as adults. I accepted the GP's suggestion that it was a virus that was lingering.

I knew for certain it must be ME about 7 months later, when we went away for a night and she had a terrible energy ‘crash’ afterwards. She was ill/‘paid’ for the trip for about a week, and it reminded me so much of my own illness. It was classic PEM. This was preceded by 5 months of going from doctor to doctor, writing letters to the Health Minister, trying to find a doctor or consultant who knew anything about pediatric ME and was equipped to assess her. It was so disheartening.

Some of the doctors, I could see in their eyes, they thought I was a hypochondriac, one said ‘she presents well today’. I felt completely unseen, unheard, I thought this is what being gaslighted feels like.

Having a child with ME, every step to get her help is a long battle. I am exhausted mentally and physically by it.

**Table 1.** Established long-term sequelae and complications by organ system for common pediatric infectious diseases according to available literature.

Parameters	Chronic fatigue	Lungs	Heart	Kidneys	Immune system	Brain	Cancers
RSV	–	✓	–	–	–	–	–
EBV	✓	–	–	–	✓	✓	✓
Measles	–	–	–	–	✓	✓	–
Poliomyelitis	✓	✓	✓	–	–	–	–
Influenza virus	✓	–	–	–	–	–	–
HIV	✓	✓	✓	✓	✓	✓	✓
*Streptococcus pyogenes*	–	–	✓	✓	–	✓	–
Dengue virus	✓	–	–	–	–	–	–
Chikungunya virus	✓	–	–	–	–	–	–
SARS-CoV-2	✓	✓	✓	✓	?	✓	–

For patients' experience refer to Box 1, for literature details refer to the Supplementary Box.

## Long covid in children

### Evidence from the literature

Carfi *et al.* first described long covid by reporting the persistence of symptoms for an average of 60 days post disease onset in hospitalized COVID-19 adult patients in Italy, in August 2020 [58]. They reported that, of 143 enrolled adults, 32% had one or two persistent symptoms and 55% had three or more for a mean of 60 days post-onset, particularly fatigue and dyspnea. This report has been later confirmed by numerous follow-up studies of COVID-19 survivors from all over the world. persistent symptoms have been reported even in younger adults and independently from the severity of acute disease [59,60]. Patients themselves have played a primary role in the description and recognition of long covid [61].

The scenario is more complex when referring to children. The description of persistent symptoms post-SARS-CoV-2 infection in children by two independent research teams in Italy [62] and Sweden [63] has initiated the long covid debate in children.

In parallel, independent parents from the UK (LongcovidKids) and France (apresJ20) started online support groups on social media to highlight that children experienced problems months after the initial infection. Similar support groups have also emerged in several countries globally. Other follow-up studies from Russia, Latvia, France, UK, The Netherlands, Germany, Spain, Australia and Switzerland have also confirmed that some children were experiencing prolonged recovery and sequelae (Table 2) [62–78]. Some of these studies reported a high prevalence of children and young people affected by one or more symptoms months after the initial infection [62–78]. Given the current lack of a definition of pediatric long covid, combined with heterogeneity and a high risk of bias in many studies, and limited access to diagnostics during the pandemic, the reported prevalence should be interpreted with caution. For example, studies without control groups report very high prevalence, which might overestimate the real burden of long covid, while those with control groups may be more appropriate estimates, although some of them have imperfect controls (e.g., children with only one negative PCR test). However, the early reports and emerging data suggests that a proportion of children might have chronic health problems deserves attention, and further investigations into etiology by pediatricians to inform care are warranted to reduce risk of chronicity and improve long-term outcomes.

**Table 2.** Main findings from studies providing details of symptoms at least 4 weeks after initial SARS-CoV-2 infection.

Study	Country	Size	Control group	Prevalence of long covid	Ref.
Buonsenso	Italy	129	No	14 (20.6%) of children interviewed >120 days after infection reported ≥3 symptoms	[62]
Brackel	The Netherlands	89	No	36% experienced severe limitations in daily function. The most common complaints were fatigue, dyspnea and concentration difficulties with 87%, 55%, and 45%, respectively	[64]
Sterky	Sweden	55	No	4 (7.2%) had multiple severe symptoms that were possibly related to COVID-19	[65]
Ludvigsson	Sweden	5	No	All children reported symptoms for 6–8 months after their clinical diagnoses	[63]
Molteni	UK	1734	1734 (based on a negative PCR nasopharyngeal test)	1.8% of the positive children experienced symptoms for at least 56 days. 0.9% in the negatively tested cohort had symptoms for at least 28 days	[66]
Osmanov	Russia	518	No	Multiple symptoms were experienced by 44 (8.4%) participants	[67]
Nogueira Lopez	Spain	72	No	Eight children reported longer lasting constitutional symptoms	[68]
Radtke	Switzerland	109	1246 (based on serology)	Four of 109 seropositive children (4%) vs 28 of 1246 seronegative ones (2%) reported at least one symptom lasting beyond 12 weeks	[69]
Say	Australia	151	No	At 3–6 months follow-up, six (4%) children mild post-viral cough, three (2%) fatigue, one (1%) had both postviral cough and fatigue	[70]
Smane	Latvia	236	142 (other infections)	95 (45.2%) COVID patients had ≥3 persistent symptoms after the 12-week cut-off point, compared with six (4.7%) of the control group	[71]
Zavala	UK	472	387 (based on a negative PCR nasopharyngeal test)	Four weeks after infection, 21/320 (6.7%) of symptomatic cases and 6/154 (4.2%) of symptomatic controls (p = 0.24) experienced on-going symptoms. Of the 65 on-going symptoms solicited, three clusters were significantly (p < 0.05) more common, albeit at low prevalence, among symptomatic cases (3–7%) than symptomatic controls (0–3: neurological, sensory and emotional and behavioral wellbeing	[72]
Borch	Denmark	37522	78,037 (not been tested positive for SARS-CoV-2, but unclear type and frequency of testing)	SARS-CoV-2 children aged 6–17 years reported symptoms more frequently than the control group (percent difference 0.8%). The most reported symptoms among pre-school children were fatigue risk difference (RD) 0.05 (CI 0.04–0.06), loss of smell RD 0.01 (CI 0.01–0.01), loss of taste RD 0.01 (CI 0.01–0.02) and muscle weakness RD 0.01 (CI 0.00–0.01). Among school children the most significant symptoms were loss of smell RD 0.12 (CI 0.12–0.13), loss of taste RD 0.10 (CI 0.09–0.10), fatigue RD 0.05 (CI 0.05–0.06), respiratory problems RD 0.03 (CI 0.03–0.04), dizziness RD 0.02 (CI 0.02–0.03), muscle weakness RD 0.02 (CI 0.01–0.02) and chest pain RD 0.01 (CI 0.01–0.01)	[73]
**Preprints**					
Blankenburg	Germany	188	1365 (based on serology)	Similar pattern of persistent symptoms in both groups	[74]
Buonsenso	UK	510	No	Parents reported a several different symptoms lasting up to 9 months	[75]
Miller	UK	175	4503 (based on serology)	The prevalence of persistent symptoms lasting ≥4 weeks in children during the second and third UK wave of the COVID-19 pandemic was1.7% overall, and 4.6% among children with a history of SARS-CoV-2 infection. Apart from children with a history of SARS-CoV2 infection, girls, teenagers and children wit	[76]
Knoke	Germany	73	45 (based on serology)	No significant differences were detected in frequency of abnormal pulmonary function (COVID-19: 12, 16.4%; controls: 12, 27.7%; OR 0.54, 95% CI 0.22–1.34) No details provided on characteristics of persistent symptoms	[77]
Stephenson	UK	3065	3739 (based on a negative PCR nasopharyngeal test)	At 3 months post-testing, 66.5% of test-positives and 53.3% of test-negatives had any symptoms, whilst 30.3% and 16.2%, respectively, had three or more symptoms	[78]

Only studies providing a detailed description of the pediatric cohort were included.

There is an ongoing debate on the etiology of long covid, and some healthcare professionals have raised concerns that some of the symptoms reported by these studies, such as fatigue, headache and mood changes might be a consequence of rigid social restrictions that children have suffered, in particular self-isolation/quarantines and school closures [79], rather than direct consequences of COVID-19 infection itself. To address this, researchers have tried to identify and characterize the real burden of long covid by performing longitudinal online surveys, either by phone or online applications, including control groups of children that supposedly have never had COVID-19. These studies [66,69] have found that many of the symptoms were frequent in both the COVID-19 positive and control groups, although generally children with documented SARS-CoV-2 infections had higher frequency and higher number of symptoms lasting for more than 12 weeks. Overall, these studies concluded that about 1% children with a recognized SARS-CoV-2 infection may experience some form of persistent sequelae for more than 3 months [80].

Although controlled studies may provide a more realistic estimation of long covid incidence, selection of controls is associated with limitations. For example, a recently published large cohort study from the UK included children that tested negative by one nasopharyngeal PCR test as controls. However, a single negative test has a very low predictive value and does not guarantee that the child has never had COVID-19. A study from Switzerland instead included children with a negative IgG test against SARS-CoV-2 as a control group [69]. Even in this case, a false negative test or rapid decay of IgG cannot firmly exclude that some controls have never been infected by SARS-CoV-2. Moreover, the use of self-completed online surveys by participants poses a risk of bias in the absence of any objective clinical assessments. This further undermines the reliability of these studies. For example, a child can have headache or fatigue for countless reasons which an online survey cannot fully elucidate. Furthermore, the increased circulation of respiratory viruses amongst young children and their exposure to several infectious agents presents an added complexity to specify their persistent symptoms as long covid. Together with the existence of asymptomatic pediatric COVID-19 cases, the limited access to or uptake of diagnostic testing, means that the true denominator of pediatric COVID-19 cases remains unknown.

Even when including control groups, many of published studies have an intrinsic, common limitation: being surveys. Although this methodology allows reaching out to a large group of individuals in a relatively short time frame, it does not collect detailed data and cannot reflect very niche and specific aspects of health status for each individual child.

So far, there are few clinical case series and reports available. Morand *et al.*, identified seven children with long covid, presenting with a similar brain hypometabolic pattern involving bilateral medial temporal lobes, brainstem and cerebellum, and the right olfactory gyrus, using PET. Similar findings have been reported in adults with long covid [81]. In our clinic, we identified perfusion defects and persistence of pro-inflammatory cytokines in an adolescent with persistent symptoms more than 6 months post-infection [82], without an alternative explanation, similar to findings in adults [83]. Bartley *et al.*, described three children with subacute neuropsychiatric impairment 2–8 weeks post-SARS-CoV2 infection. Two of them had intrathecal anti-SARS-CoV-2 antibodies, as well as intrathecal antineural antibodies, and one anti-TCF4 autoantibodies [48,84]. This patient was not tested during the acute phase, but diagnosed as an epidemiologically linked case (father with COVID-19). During the first 6 weeks following infection, this child experienced difficulties finding words, impaired concentration, difficulty completing homework, insomnia and mood lability, internal preoccupation, aggression, and suicidal thoughts. If followed up using a survey, this complex characterization may have been missed, and the child not referred for further investigations and care.

This existing literature illustrates the uncertainty in current estimates of pediatric long covid prevalence, as these studies are predominantly survey based. Therefore, this highlights the need for reliable control groups, real-time clinical investigations and further research using in-clinic diagnostics and examinations to elucidate pediatric long covid etiology and to inform better care for the sufferers.

### Evidence from personal real-world clinical practice

**Figure 1.**
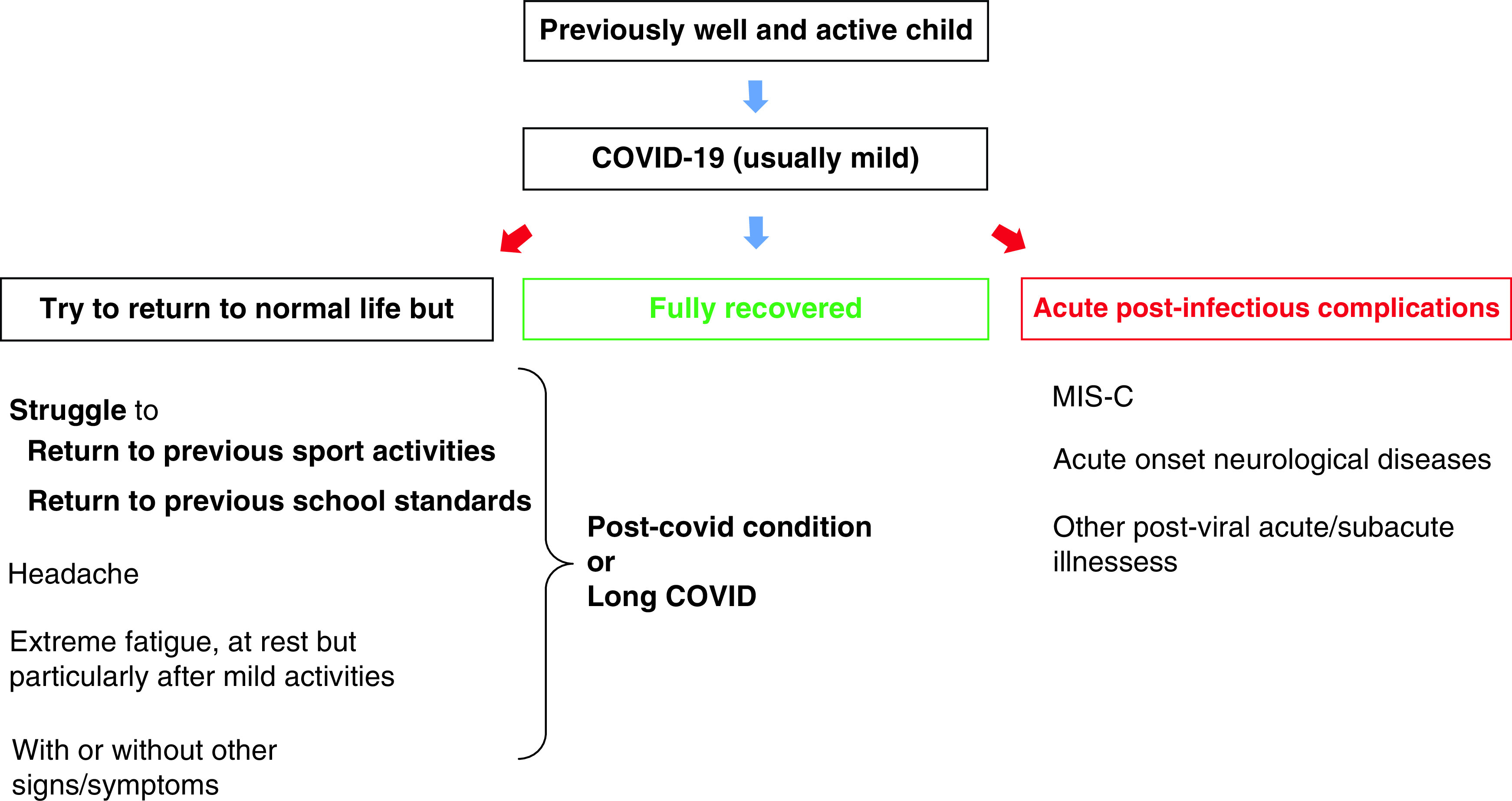
Possible outcomes of SARS-CoV-2 infection. While most children recover from acute infection (which is usually mild or asymptomatic), it is well established that some can rarely develop the multisystem inflammatory syndrome (MIS-C), or subacute sequelae (e.g., the neuropsychiatric symptoms). However, there is a subgroup of patients that apparently recover from initial infection but present a subtle clinical presentation. These children which have symptoms that impact on their return to usual activities, and usually having other signs and symptoms, are those that might better fit the diagnosis of long covid (or post-covid condition).

We started a post-covid pediatric unit at the (blinded for review) Universitario of Rome, Italy in January 2021. For over an year, we have seen an increasing number of families seeking care for their children affected by persistent symptoms following a SARS-CoV-2 infection. The most common sequelae we have observed in our clinic is persistence of severe fatigue and malaise after even mild physical or mental activities. In many cases, manifestations are so severe that they prevent children from returning to normal pre-COVID-19 activities, including school. We have seen children that used to take part in one or more sport activities including competition at a professional level that have been struggling to return to usual activity levels after COVID-19. Similarly, we evaluated children attending clinics who were suffering from headaches and fatigue while attending school. For these children, we developed a hybrid school program where they can join via online learning during days of fatigue relapse. While the majority of children recover within 6 months, some are still affected by persistent symptoms. After more than a year of experience in our center, from a clinical perspective we have defined long covid as ‘a child that, after SARS-CoV-2 infection, presents severe fatigue or post-exertional malaise that impairs the child's ability to return to routine activities, such as sport, music or education (Figure 1). Usually, a cluster of mixed symptoms are present, including cardiorespiratory (e.g. tachycardia and chest pain), gastrointestinal (e.g. nausea and frequent abdominal pain), musculoskeletal (e.g. joint and muscle pains), dermatological (e.g. rashes) and neuropsychiatric (e.g. headache, brain fog, difficulties finding words or concentrating, sleeping problems etc.) Symptoms may fluctuate and can be triggered by stress or other infections. The period of fatigue/malaise should last at least 12 weeks’. This definition is, however, complex, and difficult to translate into a research project. Our definition overlaps with the current WHO developed for adults, but provides a more specific focus on the impact of symptoms on key childhood activities, such as the ability to attend school and resume pre-COVID-19 extra-curricular activities.

**Figure 2.**
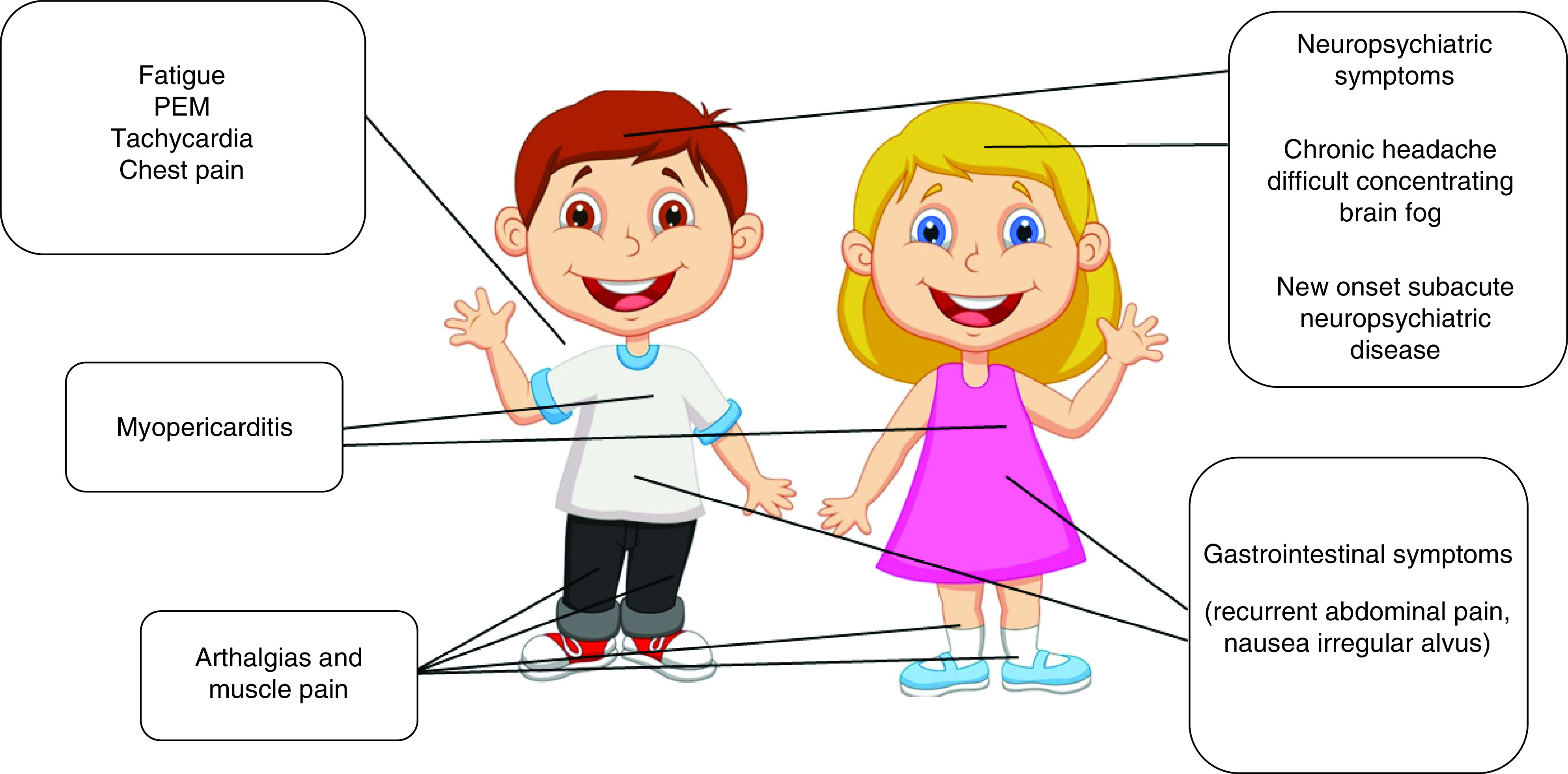
Main cluster of clinical presentation of children with long covid (personal experience). PEM: Post-exertional malaise.

**Figure 3.**
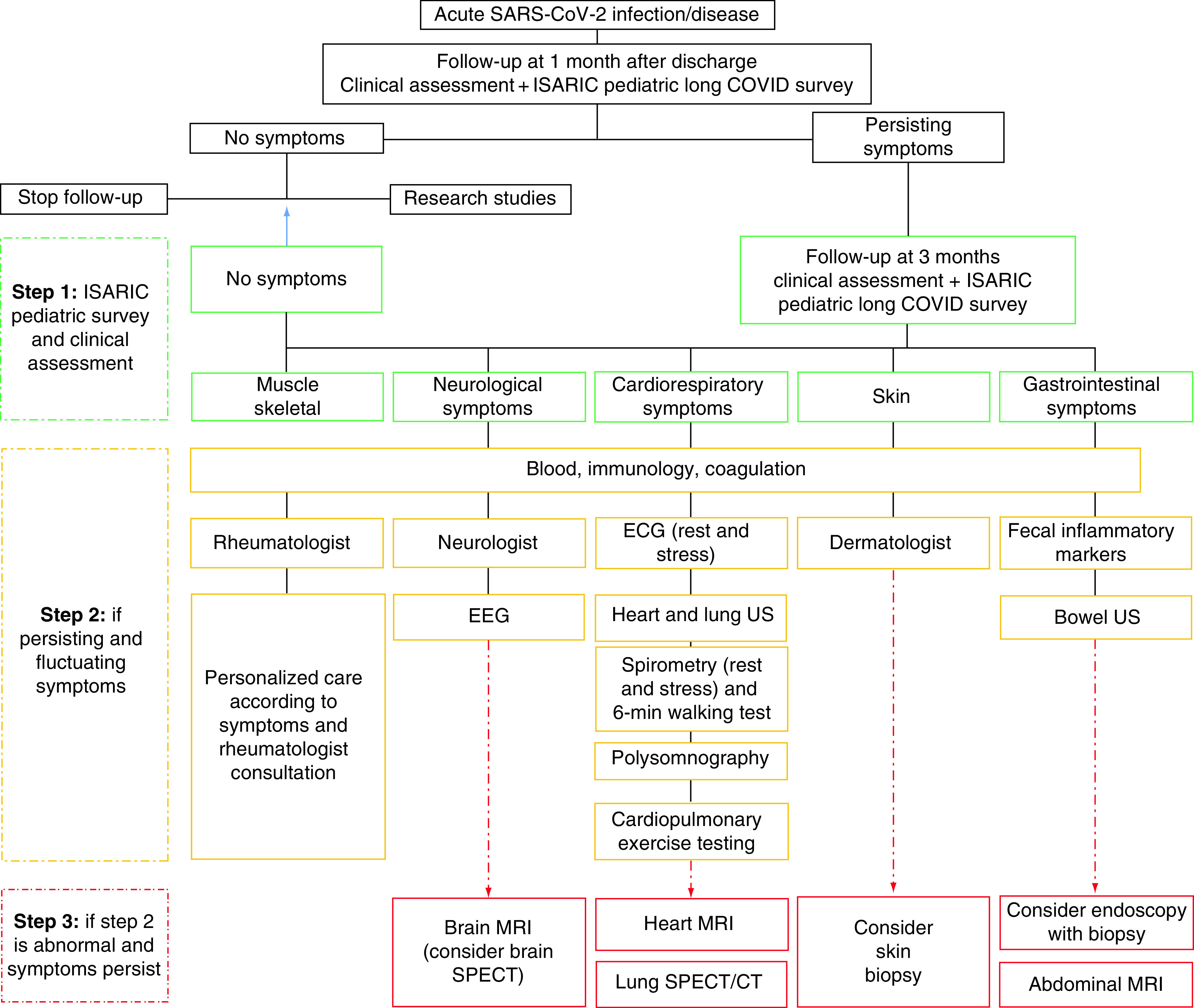
The Gemelli Hospital pediatric post-covid follow-up. EEG: Electroencephalogram. Adapted from Buonsenso *et al.* [82].

While this definition and clusters of sequelae are present in most children assessed in our clinic, it may be difficult to characterize and define using self-completion surveys not supported by further diagnostics. For children presenting with a range of symptoms, clustering is yet to be defined (Figure 2). Importantly, in light of the varied presentation seen in our clinic and in other studies, children may need a personalized diagnostic and care pathway (Figure 3). From our experience in the clinic, we have seen that children with predominantly gastrointestinal issues are those improving faster (usually within 6 months), while some of the children experiencing cardiorespiratory symptoms, general and post-exertional fatigue/malaise and severe headache have still not recovered after 12 months [82]. Some children have persistent disorders in smell and taste. These symptoms may impact the ability to perform daily activities and can lead, as a consequence, to psychological consequences. It is important to review each case individually to inform care including psychological support if needed. In our experience, it is not only important to inform the child and the families that while symptoms like fatigue and malaise are a concern and require rest, but also to ensure the child does not become socially isolated. We also advise children to attend daily activities unless severely asthenic or in pain.

### Hypotheses on mechanisms underlying long covid in adults & children

**Figure 4.**
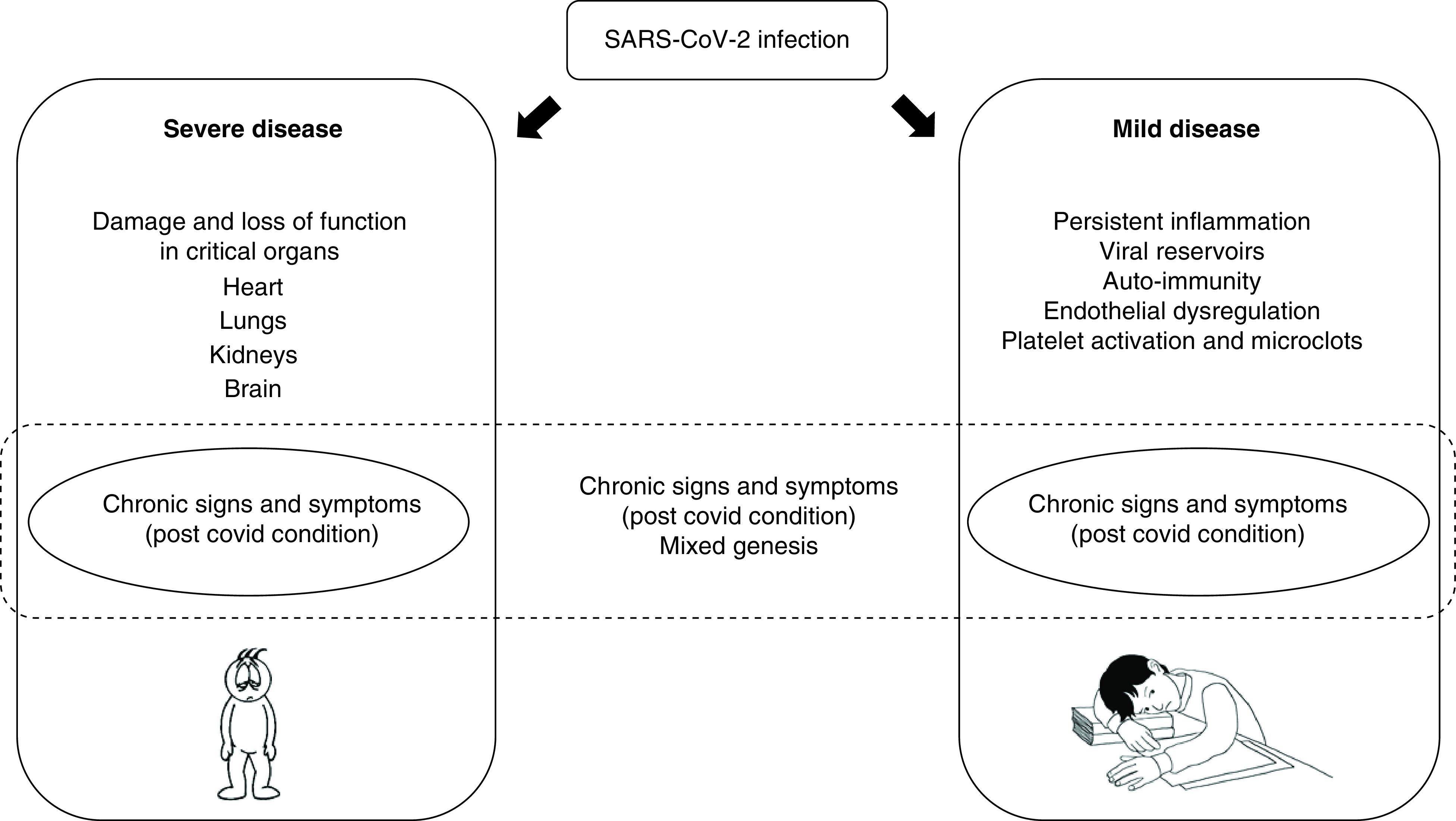
Possible mechanisms behind long covid. Organ damage due to severe acute disease can result in loss of function, which can explain chronic symptoms. Other patients, however, may have symptoms due to other less-recognized events, such as chronic inflammation, viral persistence, auto-immunity. These two main groups, however, can overlap in each patient. In general, it is possible to speculate that younger patients most probably belong to group 2, as they usually have less severe disease.

Although unclear, current hypotheses and recent evidence suggest that long covid may be divided in two main groups: a) related to organ damage during the acute illness, b) other, less well-characterized mechanisms (Figure 4).

There is clear evidence that acute SARS-CoV-2 infection, particularly in severe cases, is associated with extensive inflammatory and endothelial damage to the lungs [85], kidneys, heart, brain [86] and possibly other organs [87]. This damage can result in a loss of at least partial organ function, which has been associated with persistent symptoms. This type of long covid, related to organ damage, is likely more frequent in adults, who are at higher risk of severe COVID-19, and may be similar to sequelae seen for other acute infectious diseases (e.g., tuberculosis) [88].

In younger patients where more subtle persistent symptoms are present, the etiology may be caused by other reasons, such as autoimmunity, inflammation, viral persistence, microthrombosis and chronic endotheliopathy.

### Autoimmunity/inflammation

The relationship between viral infections and autoimmune conditions have been historically linked. Many viruses trigger autoimmune responses including the recent description of the link between Epstein–Barr virus and multiple sclerosis [89]. The traditionally accepted mechanisms behind post-viral post-acute inflammation are molecular mimicry, bystander activation (with or without epitope spreading) and viral persistence [90]. Studies have demonstrated that SARS-CoV-2 infection can induce the development of rare and uncommon autoantibodies, including autoantibodies directed against receptors (angiotensin II AT1 receptor, angiotensin [1–7] MAS) that regulate the renin–aldosterone–angiotensin (RAS) system, also found in chronic fatigue syndrome/myalgic encephalomyelitis (CFS/ME) [91].

Moreover, several *in vitro* and animal studies have shown that structural proteins derived from SARS-CoV-2 might function independently as pathogen-associated molecular pattern (PAMPs) inducing neuroinflammatory processes via pattern recognition receptor engagement in the central nervous system [92]. The SARS-CoV-2 protein has also been found to induce structurally abnormal blood clots with increased pro-inflammatory activity. This could possibly explain the mechanisms involved in coagulation disorders associated with both acute and long covid patients [93].

In the lung, acute infection recruits monocyte-derived macrophages that express genes associated with profibrotic functions. These macrophages express a profibrotic transcriptome and proteome profile that induce fibroproliferative changes in the lung [94]. Microthrombosis has also been documented [95]. How long these inflammatory profibrotic changes persist is still unknown.

The evidence of brain inflammatory changes during COVID-19 is also well established [96,97]. This is mainly related to indirect immune response effects of the immune responses, rather than direct cytopathic viral damage, including inflammation in brain endothelial cells mediated by the SARS-CoV-2 main protease Mpro [98]. The reversibility of this damage is unknown and, at least theoretically, can persist and explain some of the neurological/neuropsychiatric symptoms seen in long covid patients. Evidence of long-term brain inflammation was reported in a study by Nedham and team [99]. In a study including 250 autopsy samples they evaluated the dynamics of, and relationship between, serum markers of brain injury and markers of dysregulated host response including measures of autoinflammation and autoimmunity [99]. They found evidence of brain inflammation, documented by elevations of neurofilament light (NfL) and glial fibrillary acidic protein (GFAP) in a severity dependent manner, not only during acute disease but still persistent at follow-up 4 months later [99]. A recent radiological study using imaging studies from a UK biobank collected before and during the pandemic also identified inflammatory changes in multiple brain areas [100]. Another study also reported significant brain alterations including cortical hypometabolism in patients with isolated persistent hyposmia post SARS-CoV-2 infection [101].

Together with autoimmunity, allergies and hypersensitivity via mast cells and eosinophils are also associated with long covid. It is hypothesized that the T-helper lymphocyte type-2 (Th-2) arm of immune response which mediates allergy and helminth infection, plays a protective role in severe COVID-19 infection. However, it is also linked with immune mechanisms increasing the risk of long covid. This epitomizes the complex role of the immune system in autoimmunity and long covid [102].

### Viral persistence

Substantial evidence from independent research teams suggest that viral particles may persist in humans following many infections. In a study using a humanized mouse model, mice with chronic COVID-19 were able to elicit innate and adaptive human immune responses to SARS-CoV-2 infection up to 28 days after infection [103]. These chronically infected mice were subsequently observed with the presence of viral RNA in several tissues, chronic respiratory problems, cyclical lymphopenia and other persistent immunopathological findings possibly mediated by early recruited macrophages. Although this model mainly focuses on chronicity of infection, similar mechanisms may play a role in long covid. These findings provide preclinical data supporting hypotheses of virus persistence accounting for some symptoms experienced by patients with long covid.

These findings were confirmed in human studies, documenting viral persistence in the gastrointestinal tract >3 months follow-up [104]. Stein *et al.* analyzed autopsies of 44 patients with COVID-19 to map and quantify SARS-CoV-2 distribution, replication, and cell-type specificity across the human body, including in extrapulmonary sites. Autopsies were performed in patients who died during the acute phase or up to 7 months after infection [105]. SARS CoV-2 was found to be widely disseminated in the body despite of the severity of the acute infection. Similarly, viral persistence was also documented in red blood cells, particularly the persistence of the SARS CoV-2 S1 protein in CD16+ monocytes up to 15 months after the infection [106]. Recently, a gastrointestinal biopsy also indicated viral persistence in the gut in an adolescent infected with SARS-CoV-2 [107]. Cumulatively, viral persistence in SARS-CoV-2 patients may at least in part explain the mechanisms of long covid which result in chronic pain, fatigue, malaise, and several neurologic symptoms including brain fog and persistent ageusia/anosmia.

### COVID-19, coagulation & chronic endotheliitis

Arterial and venous thromboembolism are major causes of morbidity and mortality in patients with COVID-19 [108]. The incidence of alveolar capillary microthrombosis was nine-times higher in COVID-19-related compared to other infectious diseases [108]. These manifestations were also observed in other organs, including the heart, liver, and kidneys of COVID-19 patients [109]. Postmortem studies have confirmed an extensive microvascular thrombosis and occlusion in the lung of severe COVID-19 patients [110]. This condition has been defined as ‘thromboinflammation’ [111]. The pathological features in thromboinflammation are thrombus formation through activation of platelets, endothelial damage, coagulation cascade and activation of the immune system [108,112].

Dysregulation of these highly controlled immune responses during COVID-19 could lead to thromboembolism in patients experiencing long covid [113,114].

Cytokines such as interleukin (IL)-1, IL-2, IL-6, tumor necrosis factor (TNF) and the activation of macrophages and neutrophils play pivotal roles in the activation of the coagulation system and in the inhibition of anticoagulant mechanisms [115]. IL-1 and TNF-α induce expression the von Willebrand factor (VWF), and fibrinogen promote platelet binding in the endothelium. These cytokines suppress the anticoagulant pathways as well [116]. Moreover, activated neutrophils and macrophages express the tissue factor (TF), that initiates the so-called intrinsic blood coagulation cascade by coagulative factor VII. Also a variety of substances, including reactive oxygen species (ROS), contribute to enhancing the coagulation pathway. Platelets are another main actor in thromboinflammation. These processes occur under normal physiological condition to mediate blood clotting when required. However, inappropriate activation of this pathway during COVID-19 results in pathology in patients. Functional analysis of platelets in COVID-19 patients have demonstrated that they release a significantly higher levels of cytokines, chemokines, and growth factors during infection compared to in healthy subjects which contributes to increased thrombin, fibrinogen, VWF and factor XII [117,118]. This may explain the pathological thromboinflammation observed in COVID-19 patients.

In parallel, genetic analyzes have revealed a missense variant of ADAMTS-13 was associated with ICU hospitalization of COVID-19 patients. ADAMTS 13 is a metalloproteinase and a disintegrin that cleaves VWF inhibiting inappropriate VWF-platelet interaction [118]. Another study also demonstrated lower levels of in COVID-19 patients than the median normal reference range [118]. The higher VWF to ADAMTS-13 ratio was associated with severe COVID-19 disease [118]. Collectively, the inappropriate activation of coagulation could render possible explanations for the pathological immunothrombosis seen in COVID-19.

Under normal conditions, the endothelium is imperative for maintaining vascular integrity and barrier function. It also regulates immune response and thrombus formation through production of anti-inflammatory and antithrombotic factors, including activated protein C, tissue factor pathway inhibitor, antithrombin, nitric oxide, prostacyclin and thrombomodulin [119]. Endothelium also produces tissue plasminogen activator and urokinase PA and expresses its receptor (PAR), providing a potent fibrinolytic system. The quiescent endothelium prevents platelet adhesion by elaborating PGI2 and nitric oxide (NO), which suppresses platelet activation.

In COVID-19, endotheliopathy and endothelial blood vessel damage are commonly observed in patients [120]. SARS-CoV-2 may cause endothelial dysfunction causing immune cell infiltration, and proinflammatory cytokine production, as well as thrombosis. Moreover, by well-known ACE-2 mechanism, SARS-CoV2 infection in the endothelium might result in angiotensin II hyperactivity promoting local proinflammatory and prothrombotic signaling, which activates the kallikrein–kinin system and increases vascular permeability. The disruption of the endothelial glycocalyx which inhibits coagulation and adhesion of immune cells and platelets by shielding the endothelial wall and mediating stress-induced NO release, may lead to inflammation and thrombosis in COVID-19 [121]. Damage mediated by the infection of the endothelial membrane may encourage inflammation and thrombosis during COVID-19 due to the loss of the endothelial anticoagulant activity by its potent antithrombotic surface which expresses thrombomodulin and endothelial protein C receptor, which convert thrombin, a procoagulant into a producer of an anticoagulant; activated protein C.

Moreover, emerging evidence has identified persisting microclots in patients with long covid [122]. This may suggest that the initial thromboinflammation during COVID-19 infection persists in some patients. A working hypothesis suggests microclots in the lungs and blood circulation may lead to reduced oxygenation, which may contribute to key symptoms of fatigue and breathlessness in long covid patients. This provides a promising therapeutic rationale for supporting trials evaluating anticoagulant treatment strategies for patients diagnosed with long covid.

### Caring for children with long covid

Caring for a child with persistent symptoms after SARS-CoV-2 infection is a complex task. Firstly, because there is no available guidance to support decision-making processes and official clinical definition of pediatric long covid. Despite the lack of guidance, as health care professionals it is our duty to do our best to care for the patients, and the family as a whole.

Given the limited evidence available, this section is based on the clinical experiences of Dr B (blinded for review) (Table 3 summarizes the main characteristics of the study population followed in the outpatient setting), a pediatrician caring for children and young people in a post-covid unit, and informed by the evidence presented above, and knowledge exchange with pediatricians from other sites and countries via networking, conferences and global working groups (Figure 3).

**Table 3.** Main characteristics of children followed-up in our pediatric post-covid unit.

N (%)	169 (100%)
Age at first SARS-COV2 infection (years)	10.08
**Median age (years)**	
0–9	92 (54.4%)
10–18	77 (45.6%)
**Gender N (%)**	
Male	77 (45.6%)
Female	92 (54.4%)
**Nationality N (%)**	
Italy	165 (97.6%)
Other countries	4 (2.4%)
**Comorbidities N (%)**	
Allergic asthma	4 (2.4%)
Allergies	2 (1.2%)
Recurrent respiratory infections	2 (1.2%)
Prematurity	2 (1.2%)
D. Duchenne	1 (0.6%)
Henoch–Schonlein purpura	1 (0.6%)
Asthmatic bronchitis	1 (0.6%)
Obesity	1 (0.6%)
S. Down	1 (0.6%)
Splenomegaly	1 (0.6%)
No comorbidities	153 (90.6%)
**Acute disease severity N (%)**	
Asymptomatic	22 (13%)
Mild	133 (78.7%)
Moderate	12 (7.1%)
Severe	2 (1.2%)
**Hospital admission**	
Yes	12 (7.1%)
**PICU admission**	
Yes	4 (2.4%)
**Mean FUP (days)**	184
**Post-acute infection symptoms**	
Headache	29 (17.2%)
Dyspnea on exertion	25 (14.8%)
Muscle pain	20 (11.8%)
Chest pain	15 (8.9%)
Joint pain	14 (8.3%)
Cough	13 (7.7%)
Gastrointestinal symptoms	11 (6.5%)
Palpitations	10 (5.9%)
Altered smell	8 (4.8%)
Altered taste	7 (4.1%)
Nasal congestion/rhinorrhoea	7 (4.1%)
Rash	6 (3.5%)
Fever	5 (3.1%)
Dyspnea at rest	3 (1.8%)
Asthma	2 (1.2%)
Other: yes	55 (32.5%)
No persistent symptoms	78 (46.2%)

The first, most important step is the initial meeting with the patient and family. It is extremely important to recognize that the family is seeking medical advice and care because the child or adolescent has health problems that affect their daily life. This is a critical step, since a general assumption is that most of these children's issues are psychological. It is not unusual in our post-covid unit to meet families that have been dismissed by several healthcare professionals as having psychosomatic problems, without being heard nor properly assessed. This in itself may exacerbate the problems. Without a proper diagnostic assessment, we risk missing treatable conditions. In addition, as we have seen with other chronic diseases, dismissal of a person's symptoms and illness, commonly referred to as ‘gaslighting’, may cause great psychological damage. The increasing evidence on long covid in children as well as adults including pathophysiological mechanisms, shows the importance of an accurate assessment of each patient. It is likewise important to assess all patients, not to miss other conditions which may present with similar symptoms, e.g., common cancers, with potential serious implications. We noticed that it is important to show families our support and that we will try to understand why the child is having symptoms and how to help him. This approach also helps in building reciprocal trust. This will facilitate the assessment and recovery process. Addressing long covid issues is challenging since the evidence base is yet limited, by building reciprocal trust can help both the family and the clinician e.g. in understanding reciprocal difficulties. For the same reason, it is critical to clearly explain to the whole family the limitations of current knowledge and that it may take time and require multiple assessments to assess, diagnose and trial different support and interventions. This approach usually does not reduce, but instead tends to strengthen the family's trust. Similarly, it is important to highlight that research is advancing fast within the field, and as new knowledge is gained, further treatments and support may become available.

As previously mentioned, a standard and optimal diagnostic approach is still to be defined. In general, we support a holistic and personalized approach which, at least initially, should be aimed at excluding other conditions that can overlap with clusters of long covid symptoms. As previously shown, the clinical presentation of long covid can vary significantly from patient to patient and, inevitably, each one may require a different diagnostic pathway. If we have excluded other causes based on a clinical examination (with or without laboratory investigations), our approach is to wait and see if symptoms resolve within 12 weeks from the initial infection. Unless the patient is severely ill, since most studies have found an overall improvement as time passes.

We screen for symptom persistence and cluster them using the ISARIC global pediatric follow up case report form/survey [123]. We use this to assess the child and document information for clinical studies, a pivotal step for improving our understanding of long covid. The local guidelines that we have developed, based on our clinical experiences from our adult post-covid unit and on available evidence on possible pathophysiological mechanisms [82], have been adapted for our pediatric patients (Figure 3). Children with persisting multiple symptoms that impact on their routine activities for more than three months with other known causes excluded, are followed up with to second and third line investigations, triaged based on their main symptoms or cluster of symptoms. For those with chronic fatigue, at rest or after mild activities, we suggest cardiopulmonary exercise testing, to muscular from cardiorespiratory issues. If pathological findings are identified, then we advise on imaging, including functional studies, in consultation with the nuclear medicine specialist, since vascularization or metabolic defects may be missed using traditional imaging techniques [124]. To date, abnormal lung perfusion and brain metabolism defects have been documented in children in Italy [82] and France [81]. Since there is growing evidence that immunological events may explain some of the symptoms, extensive autoimmunity screening and assessment of immune status is pivotal. Finally, in light of emerging data on chronic endothelitis and platelet hyperactivation, it is strongly advised to assess coagulation profiles, as a minimum D-dimers, factor VIII, ristocetin co-factor, and vonWillebrand.

All physicians caring for children with post-covid sequelae are advised to stay up to date on the latest advances. We recommend engaging with local and international collaborations, both to exchange knowledge and coordinate, collaborate or align research activities. Further clinical research is pivotal to improve our knowledge, patient care and outcomes. Currently, several international networks have been implemented aiming at: i) understanding the real burden of long covid; ii) understanding the clinical presentation of long covid; iii) understanding how different centers are managing children with long covid, from a diagnostic and therapeutic perspective, with the purpose to inform, improve and standardize best available evidence based care; iiii) achieving a definition of pediatric long covid and a set of core outcomes. For example, a parent's association has developed an online resource for both family members and physicians where information and experts update current knowledge on pediatric long covid (https://www.longcovidkids.org/).

The last stage of any medical process should include a treatment strategy. For children that meet the clinical definition for long covid with no alternative diagnosis, to date, (as of 31 January 2022) there is no recognized treatment, or universally recognized standardized clinical management guidelines and a lack of interventional trials. Therefore, the management of children with long covid is particularly challenging. To identify effective treatments, novel treatment strategies should be done as part of a randomized controlled trials. However, in light of limited funding for trials and until more evidence is available, a personalized approach is necessary. Clinicians may consider common medications for specific symptoms (e.g. drugs for sleeping disorders). A number of medications might be considered for use in clinical trials based on their known biological effects, but not without a proper medical consultation (Table 4). In light of the possible biological role of oxidative stress and immune dysregulation in long covid, compounds containing multiple micronutrients which have been proved to be well tolerated and with biological effects might also warrant inclusion in trials [125–137] (Table 5). Finally, anticoagulants and SARS-CoV-2 vaccination are other considerations for clinical trials, particularly for children with more severe, long-lasting symptoms and/or with evidence of perfusion defects or microclots [122]. Anticoagulants have been used by adults with long covid, with some very preliminary positive effects found in a preprint [138], although larger confirmatory studies are needed. It has been hypothesized that vaccines may re-equilibrate immune responses and/or help with viral clearance, or divert autoimmune lymphocytes through innate cytokines [139]. Several researchers are aiming to assess clinical and laboratory changes in patients with long covid after vaccination. An early study indicated that a number of patients reported improvement of symptoms, while others relapsed. In one patient who had comorbidities and protracted SARS-CoV-2 infection for 218 days, mRNA vaccination induced a humoral and cellular immune response to SARS-CoV-2, followed by viral clearance [140].

**Table 4.** Treatments to consider for clinical trials.

Cluster/complication	Options
Gastrointestinal symptoms	Lactoferrin (?) +/- probiotics?
Headache	Paracetamol + lactoferrin and/or microelements?
Malaise–fatigue	Lactoferrin and/or microelements?
Evidence of immune dysfunction	Lactoferrin and/or microelements? Anti-inflammatory agents?
Evidence of endothelial dysfunction	Anticoagulation? Antiaggregation? Statins?
Evidence of microembolism	Anticoagulation/antiaggregation
Evidence of pericarditis	Rheumatic agents?
Sleep problems	Sleep hygiene/no alcohol/no coffee/drugs (Melatonin? Antihistamines? Others?)
Multiple symptoms	Antihistamines (overlap with mast cell activation syndrome)

‘?’ indicate that the medication represents an hypothesis to be confirmed by clinical studies.

**Table 5.** Biological effects of micronutrients and lactoferrin.

Parameter	Antiviral activity	Immune modulation	Anti inflamatory	Autoimmunity prevention	Anti-oxidant effect	Anti-thrombotic effect	Endothelial protective	Cyto protective & organ damage prevention	Anti-arrhythmic effect	Anti-depression	Microbiome
Vitamin B	–	✓	✓	–	✓	–	✓	✓	–	✓	✓
Vitamin C	–	✓	✓	?	✓	–	–	–	–	–	–
Vitamin D	✓	✓	✓	✓	✓	✓	✓	✓	✓	✓	✓
Vitamin E	–	✓	✓	?	✓	✓	✓	✓	–	–	✓
Magnesium	–	✓	✓	?	✓	✓	✓	✓	✓	?	–
Selenium	✓	✓	✓	✓	✓	✓	✓	✓	?	?	?
Zinc	✓	✓	✓	✓	✓	–	✓	–	–	✓	?
Phyto chemicals	✓	✓	✓	✓	✓	✓	✓	✓	✓	✓	✓
Lactoferrin	✓	✓	✓	–	✓	–	–	–	–	–	✓

## Conclusion

**Figure 5.**
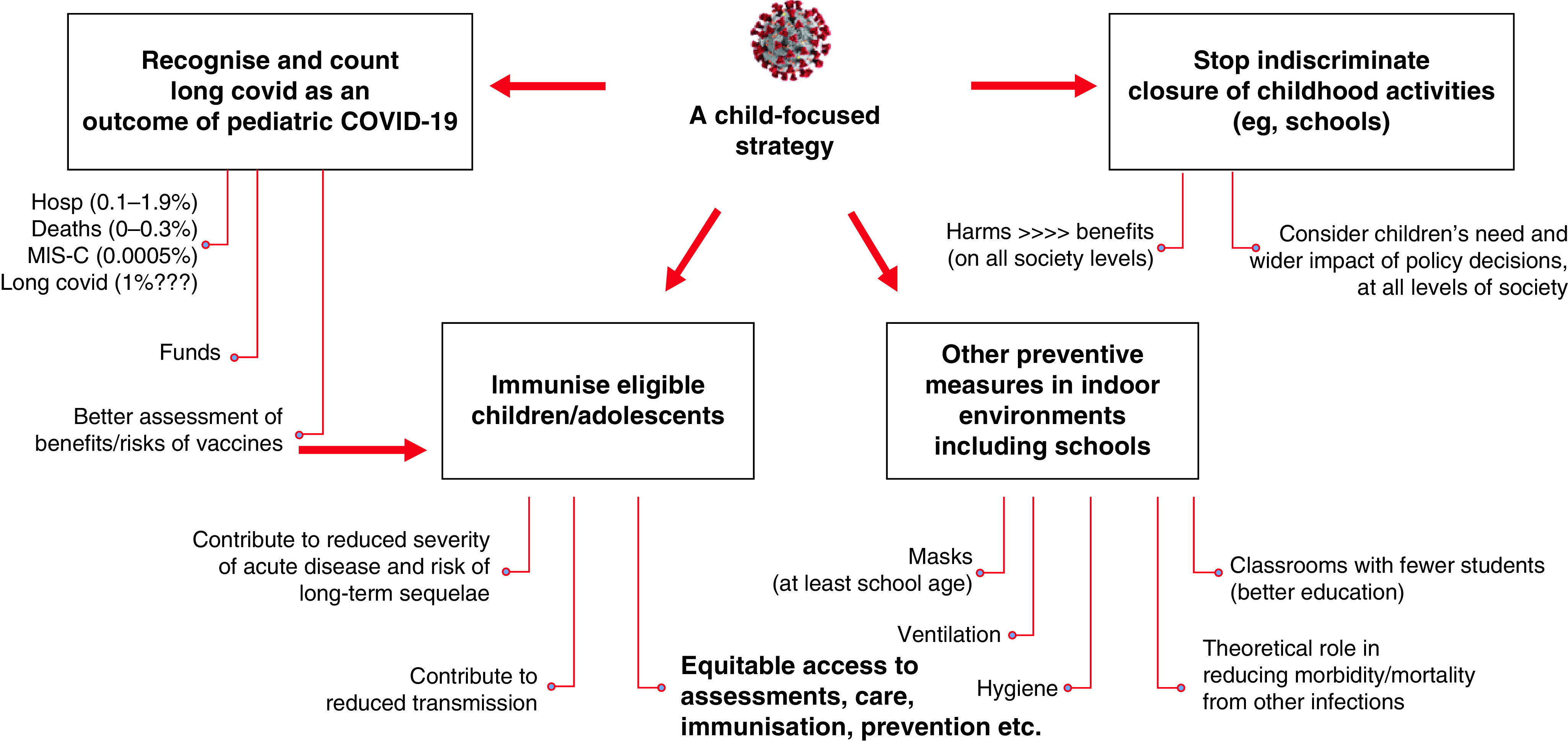
Implications of counting long covid as a possible outcome of SARS-CoV-2 infection on the risk/benefits assessment of preventive strategies.

There is increasing, emerging evidence that children can be affected by long-term SARS-CoV-2 sequelae and post-covid condition, although at a lower rate compared to adults. Studies show that most children recover spontaneously within the first six months, but not all. Studies indicate that the risk is higher in older children. Even for those that recover, long covid may have implications in terms of socializing and education and they may need additional support to catch up with their peers. Moreover, with our increased understanding of potential pathological mechanisms behind long covid, it is important that long term sequelae in children are not dismissed as psychological problems. It is important to offer these children comprehensive diagnostic assessments, care and support. Also, recognizing long covid as a possible outcome of SARS-CoV-2 infection (in the general population, and in children), will enhance our ability to assess benefits and risks of preventive measures, such as vaccination (Figure 5). Funding for research into etiology of long covid and interventional treatment trials including children and adults is urgently needed to inform treatment strategies and improve long term covid patient outcomes. Understanding long covid, its pathogenesis, diagnostic pathways and best management, offers a unique opportunity to also improve our understanding of other post-viral syndromes. Ultimately, this will have a positive impact on the health of a very large number of people with chronic, undiagnosed diseases, struggling to access appropriate care.

## Future perspective

Despite recent advances in the understanding of long term complications after acute infectious diseases, the pathogenesis of these late effects are still unknown. Also, while non-specific conditions like CFS/ME have been historically linked with viral infections, causative mechanisms have never been demonstrated. We hypothesize that the current interest in long covid will help the scientific community in better understanding, recognition and management of long-term sequelae of infections. More specifically, we speculate that infections, in genetically predisposed patients, can lead to immune dysregulation, chronic inflammatory processes, neuro-inflammation. Viral persistence, in terms of particles, can also play a role in triggering such mechanisms. Hopefully, new biomarkers can help in recognize these children and guide the development of new generation immune treatments.

**Executive summary**

**Lessons from historical infectious diseases**

- Long-term complications after acute infections are well established. Measles, bronchiolitis, poliomielities, or infections caused by HIV, EVB, HSV Influenza and other viruses, are all linked with well characterized late complications.

**Current knowledge of long covid**

- Long covid in adults is well characterized.
- Pediatric long covid has been increasingly described by several authors worldwide, although its incidence is unclear.
- Immunological and inflammatory mechanisms, as well viral persistence and coagulation issues can play a role in its pathogenesis.
- There are still uncertainties on the best diagnostic and therapeutic approach.
- We discuss the experience of our post-covid pediatric unit in Rome, summarize our diagnostic pathways and the rational behind it, and also mention currently evaluated therapeutic options.
