# Supplementary material for: Long-term outcomes of pediatric infections: from traditional infectious diseases to long covid
Source: Future Microbiol. 2022 Mar 10:10.2217/fmb-2022-0031. doi: 10.2217/fmb-2022-0031 (PMC8910780; doi:10.2217/fmb-2022-0031)
Supplement: Supplementary file 1 [file supplementary_material.docx]

**Supplementary material**

**Supplementary box. Longer-term outcomes of infections: lessons learnt so far**

| ***Respiratory Syncytial Virus (RSV)***  RSV is the leading etiological agent of acute bronchiolitis and one of the leading causes of hospitalization and mortality in children (4, 5). Although mechanisms are not fully established, several longitudinal studies have linked bronchiolitis in infancy with the development of recurrent wheezing and asthma, often requiring chronic inhaled therapy or multiple admissions, for years (6). In a recent study, including follow up of 266 children with bronchiolitis , 36 (13.5%) developed recurrent wheezing (RW) group, 65 (24.4%) were diagnosed with asthma (AS), while the remaining 165 (62.1%) recovered fully (6). Importantly, Zhang *et al*., found that the relative abundance of *Haemophilus, Moraxella,* and *Klebsiella* was higher in infants who later developed recurrent wheezing than in those who did not (LDA score >3.5). Airway levels of LPS (P = .003), CXCL8 (P = .004), CCL5 (P = .029), IL-6 (P = .004), and IL-13 (P < .001) were significantly higher in infants who later developed recurrent wheezing than in those who did not. Moreover, high airway abundance of *Haemophilus* was associated with higher CXCL8 (r = 0.246, P = .037) levels. Abundance of Moraxella was associated with higher IL-6 level (r = 0.236, P = .046) and IL-10 level (r = 0.266, P = .024) (7). These data suggests that imbalances in host microbiota during an acute infection may impact on risk of longer term sequelae. Other post-infectious RSV immune dysregulations have been described: Maleewan Kitcharoensakkul *et al.,* found lower TNF-α production by *in vitro* stimulated CD4+ T cells during severe RSV bronchiolitis in children who subsequently developed recurrent wheezing. This data supports the role of CD4+ T cell immunity in the development of post-acute RSV wheezing in these children (8, 9). In addition, different dysregulated interferon pathways have also been implicated in post-bronchiolitis wheezing (8, 9).  ***Epstein-Barr virus (EBV)***  Epstein-Barr virus (EBV) is a double-stranded DNA virus belonging to the Herpes family. It is a common infectious agent, found in approximately 95% of the world’s population. Primary infection with EBV is more frequent during childhood, usually asymptomatic or presenting with mild symptoms. generally and causinges a mild infection, usually asymptomatic. which usually presents with no symptoms. When primary infection occurs during adolescence, it leads to infectious mononucleosis (IM) in 30-70% of cases, with risk of (10)*.* However, several, well-established post acute complications are well established.  EBV is transmitted by saliva and initially infects epithelial cells in the oropharynx and nasopharynx. After which wards, EBV enters the underlying tissues and infects B-cells (10, 11)*.*  Latently infected B cells in peripheral blood are present for years after acute EBV infection. Each cell carries approximately two to five copies of an intact circular (latent, episomal) viral genome (10, 12).  EBV has a unique biologic activity that transforms B-lymphocytes into lymphoblastoid cells that have the potential to proliferate autonomously in vivo. A This process might be responsible for the pathogenesis of neoplastic diseases associated with the virus *(12).*  **Lymphoproliferative cancers** are the most well established late-onset complication ofto EBV infection. As EBV preferentially infects B- cells via the CD21 receptor, it has a strong association with B-lineage-lymphoproliferative diseases or lymphomas, such as post-transplant lymphoproliferative disorders, Hodgkin lymphoma, Burkitt lymphoma and EBV positive diffuse large B-cell lymphoma (13, 14)*.*  It also infects T- or natural killer (NK)-lineage cells at a lower frequency and it is associated with T- or NK-cell malignancies, such as extranodal NK/T cell lymphoma and aggressive NK-cell leukaemia (15)*.*  EBV infection is also associated with **epithelial cell malignancies** such as **nasopharyingeal carcinoma (NPC) and gastric carcinoma**. The latent EBV infection in pre-invasive nasopharyngeal epithelium might be an early stage of NPC pathogenesis. *Campion NJ et. al* showed that p16 inactivation and Cyclin D1 overexpression appear to be important early events to allow EBV latency in nasopharyngeal cells (16)*.* Moreover, some studies suggest that dysregulated NF-κB (Nuclear Factor Kappa-light-chain-enhancer of activated B cells) signaling may contribute to the establishment of latent EBV infection in NPC. Stable EBV infection and the expression of latent EBV genes are responsible for transformation of pre-invasive nasopharyngeal epithelial cells to cancer cells through multiple pathways (17, 18)*.*  **Myalgic encephalomyelitis/chronic fatigue syndrome (ME/CFS)** is a chronic multisystem disease characterized by at least six months of fatigue and various other symptoms, including headache, sore throat, muscle pain, joint pain, muscle weakness, post-exertional malaise, orthostatic intolerance, cognitive problems, anxietysleep abnormalities, anxiety and depression. As demonstrated in a recent study by [*Evelina Shikova*](https://pubmed.ncbi.nlm.nih.gov/?term=Shikova+E&cauthor_id=32129496)*et al.* EBV infection is a well-known trigger of CFS (19)*.* According to *Ben Z. Katz et al*. among 301 adolescents with IM, 11-13% are reported to have CFS six months after EBV infection. Several mechanisms have been proposed to be involved in the development of ME/CFS after EBV infection. *Ruiz-Pablos et al.* recently suggest that the pathogenetic mechanism underlying ME/CSF and EBV is the ability of EBV-infected latent cells to escape cellular surveillance. They also hypothesize that within ME/CFS there is a subgroup of patients with DRB1 and DQB1 alleles that could confer greater susceptibility to EBV (20-22)*. Kerr J.* in his studies reported upregulation of Early Growth Response genes (EGR1, EGR2, EGR3) occurred in ME/CFS patients with a raised level of EBV virus induced gene 2 (EBI2) mRNA, suggesting that expression of these four genes in peripheral blood mononuclear cells (PBMC) of ME/CFS patients was linked with EBV infection/reactivation (20-22)*.* Neuroinflammation is also involved in ME/CFS pathogenesis (23).  EBV could be associated also with **autoimmune diseases**, such as rheumatoid arthritis, systemic lupus erythematosus, multiple sclerosis (24), inflammatory bowel diseases, autoimmune thyroiditis, insulin-dependent diabetes mellitus, Sjögren’s syndrome, autoimmune liver diseases, systemic sclerosis and myasthenia gravis (25-28)*.* One of the main mechanisms underlying the link between EBV infection and autoimmunity may be molecular mimicry *or* by ‘bystander activation’ (25-28)*.*  **Alice in Wonderland syndrome** is described both during acute infections and during EBV reactivations (28)**.**  Exposure to EBV has also been associated with attachment **anxiety, psychotic experiences** in adolescence, and **cognitive impairments** in some individuals (29-31). *Dickerson F. et. al* found that individuals with schizophrenia had elevations in the levels of antibodies to EBV virions as compared to the control population (29-31)*.* EBV is a known neurotropic infectious agent. The human brain continues to develop during childhood and early adulthood; hence, infection during this period could potentially increase the risk of neurological abnormalities. Possible pathophysiological mechanisms include inflammatory cytokines affecting the brain after the activation of the innate immune system (25). Last, growing evidence suggest that EBV also play a primary role in the pathogenesis of multiple sclerosis (24).  ***Streptococcus pyogenes***  *Streptococco Pyogenes, also known as group A Streptococcus (GAS) is a gram positive bacterium which frequently causes human infections such as pharyngitis and* Scarlet fever, or more severe disease like necrotizing fasciitis and streptococcal toxic shock syndrome. However, GAS is much well defined for its post-infectious sequeale, suche as **Acute Rheumatic Fever, Post-streptococcal acute glomerulonephritis or Pediatric Autoimmune Neuropsychiatric Disorders** associated With Streptococcal Infections (PANDAS). In all cases, a complex interaction between Streptococcus Pyogenes and a susceptible host, in a stage set by enviromental factors, seem to contribute a role in their development. Importantly, autoimmune responses have been documented in all these syndromes (32, 33).  ***Dengue virus and chickungunia***  Dengue fever is a mosquito-borne illness that occurs in tropical and subtropical areas of the world. In a prospective study from Singapore 127 patients hospitalized for dengue fever were interviewed by telephone 2 months after the acute infection and compared with non-dengue patients with an acute febrile illness; post-infectious fatigue was found in 25% of patients, Dengue patients had a significantly higher risk of fatigue at 2 months after the acute infection (RR 4.93 [95% confidence interval {CI} 2.3 to 10.4], p<0.001) (34).  Also, long-term sequelae such as arthralgia, arthritis, depression and alopecia after chikungunya’ infection have been described and negatively impact on Quality of Life (35). Moro et al. evidenced that persistent arthralgia after acute infection varied from 5% (24/509) at a time-interval of 24 months, to 60.8% (138/227) at a time-interval of 12-13 months (36). Depression and/or depressed mood were reported as a longterm consequence of a Chikungunya infection. Two groups demonstrated a significant difference in the occurrence of depression between seropositive and seronegative persons, 17 and 24 months after infection respectively: 13% (25/199) vs. 5% (10/199) and 14.7% (75/512) vs. 8.0% (47/582), respectively, with a relative risk of 2.5 (CI 1.5-4.1)5 and a crude prevalence ratio of 1.8 (CI 1.2-2.7) (37).  ***Poliomyelitis***  Poliomyelitis has been one of the scariest and most acutely debilitating infections in the 20^th^ century affecting millions of people before the introduction of an effective vaccination. The poliovirus is transmitted by person-to-person spread mainly through the fecal-oral route or, less frequently, by a common vehicle. Although initial symptoms are fever, fatigue, headache, vomiting, stiffness of the neck and pain in the limbs, one in two hundred infections leads to irreversible paralysis (usually in the legs) which can cause death in case of involvement of respiratory muscles (38). Importantly, years after infection several survivors develop post-polio syndrome, characterized by new, persistent, and progressive muscle weakness, atrophy, limb fatigability, myalgia, arthralgia, and dysphagia, but also as generalized fatigue, which typically has a considerable impact on the patients’ quality of life (39).  ***Measles***  Measles shares several similarities with SARS-CoV-2, being both RNA viruses with an airborne transmission route and both can cause a systemic illness ranging from a mild to severe and even fatal disease in all age groups. Moreover, although pathophysiological mechanisms are not yet completely understood, the acute and long-term consequences of measles infection are clinically well characterized and widely accepted since decades (40), representing an useful model to understand the long-term consequences of viral infections, including Covid-19.  Focusing on post-acute complications, historical epidemiological and clinical studies (41) showed that measles weakens the immune system causing a measles-induced immune suppression predisposing to secondary infections (42). More in details, epidemiological studies clearly linked non-measles infectious disease mortality in high-income countries to measles incidence, in both the pre- and post-vaccine eras (43). Recent immunological studies are providing rational for these findings (44). It is now accepted that measles infection causes depletion of T and B lymphocytes (43) and, more recently, an important study showed that measles caused elimination of 11 to 73% of the antibody repertoire across individuals in a cohort of 77 unvaccinated children assessed before and two months after infection using VirScan, an assay that tracks antibodies to thousands of pathogen epitopes in blood (45). Interestingly, these immune system effects were not observed in infants vaccinated against MMR (measles, mumps, and rubella) and recovery of antibodies was detected after natural re-exposure to pathogens.  Unfortunately, measles also has well-known long-term neurological complications occurring within days to years after the infection, including primary measles encephalitis, acute post-infectious measles encephalomyelitis, measles inclusion body encephalitis, and subacute sclerosing panencephalitis (SSPE) (46). In all cases, complex interactions between the virus and immune system, or the persistence of the virus in the central nervous system, are implicated. In sclerosing panencephalitis, the virus isolated from brain tissue of patients with SSPE is missing 1 of the 6 structural proteins, the matrix or M protein, which is responsible for assembly, orientation, and alignment of the virus in preparation for budding during viral replication (47). This immature virus may be able to reside, and possibly propagate, within neuronal cells for long periods and stimulate chronic inflammatory changes. The fact that most patients with SSPE were exposed at a young age suggests that immune immaturity is involved in pathogenesis (47). Importantly, these sequelae develop years after initial infection and diagnosis is challenging since most usual assessments are normal, including cerebrospinal fluid changes and brain magnetic resonance, while only biopsies may mostly show some clinical clues. Of note, these patients usually have evidence of intratecal production of measles IgG antibodies. This concept is extremely relevant, since recently a case series of three children with subacute neuropsychiatric symptoms were found to have intrathecal anti–SARS-CoV-2 antibodies as well as intrathecal antineural antibodies (48) and, overall, neurological manifestations were found to be relatively common in children with Covid-19 or multisystem inflammatory syndrome (49).  ***Influenza***  A recent study aiming to assess long covid in a cohort of Covid-19 patients compared with influenza patients found that symptoms like abnormal breathing, fatigue/malaise, chest/throat pain, headache, abdominal symptoms, myalgia, cognitive, and anxiety/depression, although more frequent in Covid-19 survivors, were frequent also in flu ones (50). Chen et al performed a 2-year follow-up of a cohort of fifty-six influenza A (H7N9) survivors, showing that long-term lung disability and psychological impairment in H7N9 survivors persisted at 2 years after discharge from the hospital (51). A similar finding was reported by Whang et al in different cohorts of patients suffering from H7N9 influenza during different outbreaks (52). Also, a higher risk in severe cardiovascular events has been found in influenza survivors (53, 54). These findings are not surprising, since similar chronic sequelae have been reported even after then Spanish Flu in 1918 (55).  In addition, non-neurotropic H3N2 Influenza virus infection in mice was able to induce neuroinflammatory responses generating neuroinflammation in the hippocampus, impaired spatial learning, microglia activation, rise in amyloid-β plaques load and cognitive, and reduced CA1 hippocampal dendritic spine density (56, 57).  ***HIV***  The massive long-term impact of HIV in humans is very well established and cannot be summarized in a single chapter. Being so well characterized, it is important to remember that even HIV begins as an either asymptomatic infection of with a flu-like syndrome, which, only years later, fully manifests its impact on the immune system of the infected individual. |
| --- |
